# CuATSM improves motor function and extends survival but is not tolerated at a high dose in *SOD1*^*G93A*^ mice with a C57BL/6 background

**DOI:** 10.1038/s41598-021-98317-w

**Published:** 2021-09-29

**Authors:** Jeremy S. Lum, Mikayla L. Brown, Natalie E. Farrawell, Luke McAlary, Diane Ly, Christen G. Chisholm, Josh Snow, Kara L. Vine, Tim Karl, Fabian Kreilaus, Lachlan E. McInnes, Sara Nikseresht, Paul S. Donnelly, Peter J. Crouch, Justin J. Yerbury

**Affiliations:** 1 Illawarra Health and Medical Research Institute, Wollongong, NSW Australia; 2 School of Chemistry and Molecular Bioscience, Molecular Horizons, Faculty of Science, Medicine and Health, University of Wollongong, Wollongong, NSW 2522 Australia; 3 School of Medicine, Western Sydney University, Campbelltown, NSW 2560 Australia; 4 Department of Biochemistry and Pharmacology, University of Melbourne, Parkville, VIC 3010 Australia; 5 School of Chemistry and Bio21 Molecular Science and Biotechnology Institute, University of Melbourne, Parkville, VIC Australia

**Keywords:** Amyotrophic lateral sclerosis, Drug safety

## Abstract

The synthetic copper-containing compound, CuATSM, has emerged as one of the most promising drug candidates developed for the treatment of amyotrophic lateral sclerosis (ALS). Multiple studies have reported CuATSM treatment provides therapeutic efficacy in various mouse models of ALS without any observable adverse effects. Moreover, recent results from an open label clinical study suggested that daily oral dosing with CuATSM slows disease progression in patients with both sporadic and familial ALS, providing encouraging support for CuATSM in the treatment of ALS. Here, we assessed CuATSM in high copy *SOD1*^*G93A*^ mice on the congenic C57BL/6 background, treating at 100 mg/kg/day by gavage, starting at 70 days of age. This dose in this specific model has not been assessed previously. Unexpectedly, we report a subset of mice initially administered CuATSM exhibited signs of clinical toxicity, that necessitated euthanasia in extremis after 3–51 days of treatment. Following a 1-week washout period, the remaining mice resumed treatment at the reduced dose of 60 mg/kg/day. At this revised dose, treatment with CuATSM slowed disease progression and increased survival relative to vehicle-treated littermates. This work provides the first evidence that CuATSM produces positive disease-modifying outcomes in high copy *SOD1*^*G93A*^ mice on a congenic C57BL/6 background. Furthermore, results from the 100 mg/kg/day phase of the study support dose escalation determination of tolerability as a prudent step when assessing treatments in previously unassessed models or genetic backgrounds.

## Introduction

Amyotrophic lateral sclerosis (ALS) is a neurodegenerative disease involving deterioration of both upper and lower motor neurons. Disease progression is rapid, with patients developing progressive muscle weakness, subsequently leading to respiratory failure and death typically within 2–3 years^1^. Currently, there are two FDA approved therapeutics for ALS; riluzole and edaravone. Both of these drugs provide minimal therapeutic benefit^2–5^ highlighting a need for more effective treatment options.

A large majority of ALS cases have no hereditary origin and are classified as sporadic ALS (sALS); whereas, approximately 10% of cases are inherited and classified as familial ALS (fALS). The first gene to be associated with ALS was *SOD1*, which now has > 160 identified mutations linked with ALS^6^. *SOD1* encodes for the Cu/Zn superoxide dismutase (SOD1) enzyme that functions as a free radical scavenger by converting superoxide (O_2_^**·**−^) to hydrogen peroxide and molecular oxygen^7^. Many fALS *SOD1* mutations do not alter the activity of the enzyme, which remains similar to wild-type SOD1^8^. In addition, *SOD1* knock-out models do not exhibit ALS pathology, indicating loss-of-function is not sufficient to cause ALS pathology^9^. Conversely, mice overexpressing wild-type *SOD1* reportedly display neuromuscular deficits, further indicating a gain-of-function mechanism as the mode of SOD1 toxicity^10,11^. Moreover, transgenic mice overexpressing mutant *SOD1* (i.e. *D90A, G85R, G37R, G93A*) display ALS-like symptoms and pathology to varying degrees and severity (for review see^12^). However, all fALS-associated *SOD1* mutations share the unifying feature of promoting SOD1 to misfold, leading to the formation of insoluble protein aggregates/inclusions in motor neurons and proximal cells^13,14^. The folding pathway of SOD1 involves several post-translational modifications to achieve its final, homodimeric, functional form. In its native conformation, SOD1 is thermodynamically stable and remains catalytically active even in high concentrations of denaturant^15,16^. A critical step in the proper maturation of SOD1 structure is the addition of a redox active copper ion. In addition to SOD1 stability, copper is an essential element with its bioavailability and in vivo homeostasis tightly controlled. Furthermore, *SOD1* rodent models exhibit copper dyshomeostasis^17–20^. Copper bioavailability was recently reported to be altered in sALS post-mortem tissue, and associated accumulation of iron in the motor cortex^21^ suggesting that changes to copper metabolism may not be specific to *SOD1* fALS^22^.

CuATSM (diacetyl*bis*(*N(*4)-methylthiosemicarbazonato)copper(II)) is a stable (*K*_*a*_ = 10^18^), low molecular weight, charge neutral, lipophilic, compound that is able to cross the blood–brain barrier^17,18,23,24^. CuATSM has neuroprotective activity in animal models of diverse neurodegenerative disorders, including cerebral ischemia, neuroinflammation, Parkinson's disease and ALS^23,25–27^. Preclinical investigation of CuATSM treatment (30–200 mg/kg/day) in numerous *SOD1* ALS mouse models has demonstrated the ability of CuATSM to significantly delay the onset of ALS symptoms, reduce weight loss and extend lifespan (Table 1), with no reported adverse side effects attributed to CuATSM treatment. In light of these encouraging preclinical results, the tolerability and efficacy of CuATSM in patients with ALS is now being assessed in a multicentre, randomized, double-blind, placebo controlled study in Australia (NCT04082832 and NCT02870634).

**Table 1:** Therapeutic outcomes for CuATSM across multiple mouse models of amyotrophic lateral sclerosis.

Mouse model	Genetic background	Commencement of treatment	Daily dose (mg/kg)	Administration route	Increase in survival	Body weight	Motor function	Neurological score	Study
Low copy *SOD1-G93A*	Congenic; C57BL/6	140 days (presymptomatic)	30 (5 days per week)	Oral	14%	Delayed onset	Delayed onset/slowed progression	Delayed onset	23
Low copy *SOD1-G93A*	Congenic; C57BL/6	200 days (symptomatic)	30 (5 days per week)	Oral	10%	NR	Slowed progression	NR	23
High copy *SOD1-G37R*	Congenic; C57BL/6	40 days (presymptomatic)	10 (7 days per week)	Oral	8%	NR	Delayed onset/slowed progression	NR	24
High copy *SOD1-G37R*	Congenic; C57BL/6	40 days (presymptomatic)	30 (7 days per week)	Oral	18%	NR	Delayed onset/slowed progression	NR	24
High copy *SOD1-G37R*	Congenic; C57BL/6	40 days (presymptomatic)	60 (7 days per week)	Oral	26%	NR	Delayed onset/slowed progression	NR	24
High copy *SOD1-G37R*	Congenic; C57BL/6	149 days (symptomatic)	60 (7 days per week)	Oral	12%	NR	Slowed progression	NR	24
High copy *SOD1-G37R*	Congenic; C57BL/6	40 days (presymptomatic)	30 (7 days per week)	Oral	18%	NR	Delayed onset/slowed progression	NR	17
High copy *SOD1-G93A*	Mixed; B6SJL	5 days	200 (7 days per week)	Transdermal	25%	Slowed progression	NR	Delayed onset	18
High copy *SOD1-G93A*	Mixed; B6SJL	50 days	200 (7 days per week)	Transdermal	19%	Slowed progression	NR	Delayed onset	18
High copy *SOD1-G93A* × *CCS*	Mixed; B6SJL	Prenatal	60 (7 days per week)	Transdermal	2800%	Slowed progression	NR	NR	18
High copy *SOD1-G93A*	Mixed; B6SJL	50 days (presymptomatic)	100 (7 days per week)	Oral	9%	NR	Delayed onset/slowed progression	Delayed onset	20
High copy *SOD1-G93A*	Mixed; B6SJL	50 days (presymptomatic)	30 (7 days per week)	Oral	*Improved survival by 5.5 days	Slowed progression	NR	Delayed onset	45
Neurotoxin; β-sitosterol β-d-glucoside	Outbred;CD-1	63 days	30 (5 days per week)	Transdermal	NR	No significant difference	Slowed progression	Improved leg extension reflex	47

*Trends toward extended lifespan in mice treated with CuATSM compared to vehicle-treated mice, although the effects were not statistically significant. *NR* not reported.

In the current study, we tested an oral dose of 100 mg/kg/day CuATSM in a high copy number *SOD1*^*G93A*^ mouse model on a congenic C57BL/6 background. This dose in this specific model has not been assessed previously. CuATSM was suspended in a standard suspension vehicle (0.9% w/v NaCl, 0.5% w/v Na-carboxymethylcellulose, 0.5% v/v benzyl alcohol, 0.4% v/v Tween-80) and administered by oral gavage twice daily from 70 days old. We observed that at this dose in *SOD1*^*G93A*^ mice maintained on a C57BL/6 background, a subset of CuATSM-treated mice exhibited clinical signs of toxicity including hunched posture, piloerection and were hypoactive following 3–51 days of treatment. Subsequently, mice remaining in the cohort were given a 1-week wash-out period and recommenced CuATSM treatment at 60 mg/kg/day, which consequently delayed disease progression and prolonged lifespan.

## Results

### Oral CuATSM administration (100 mg/kg/day) induces clinical signs of toxicity and weight loss in a subset of *SOD1*^*G93A*^ mice on C57BL/6 background

To investigate the therapeutic efficacy of CuATSM on ALS progression, age- and gender-matched *SOD1*^*G93A*^ mice (C57BL/6 background) were treated twice daily with vehicle or CuATSM (total dose of 100 mg/kg/day) from 70 days old (presymptomatic with no clinical symptoms or motor impairment).

**Figure 1:**
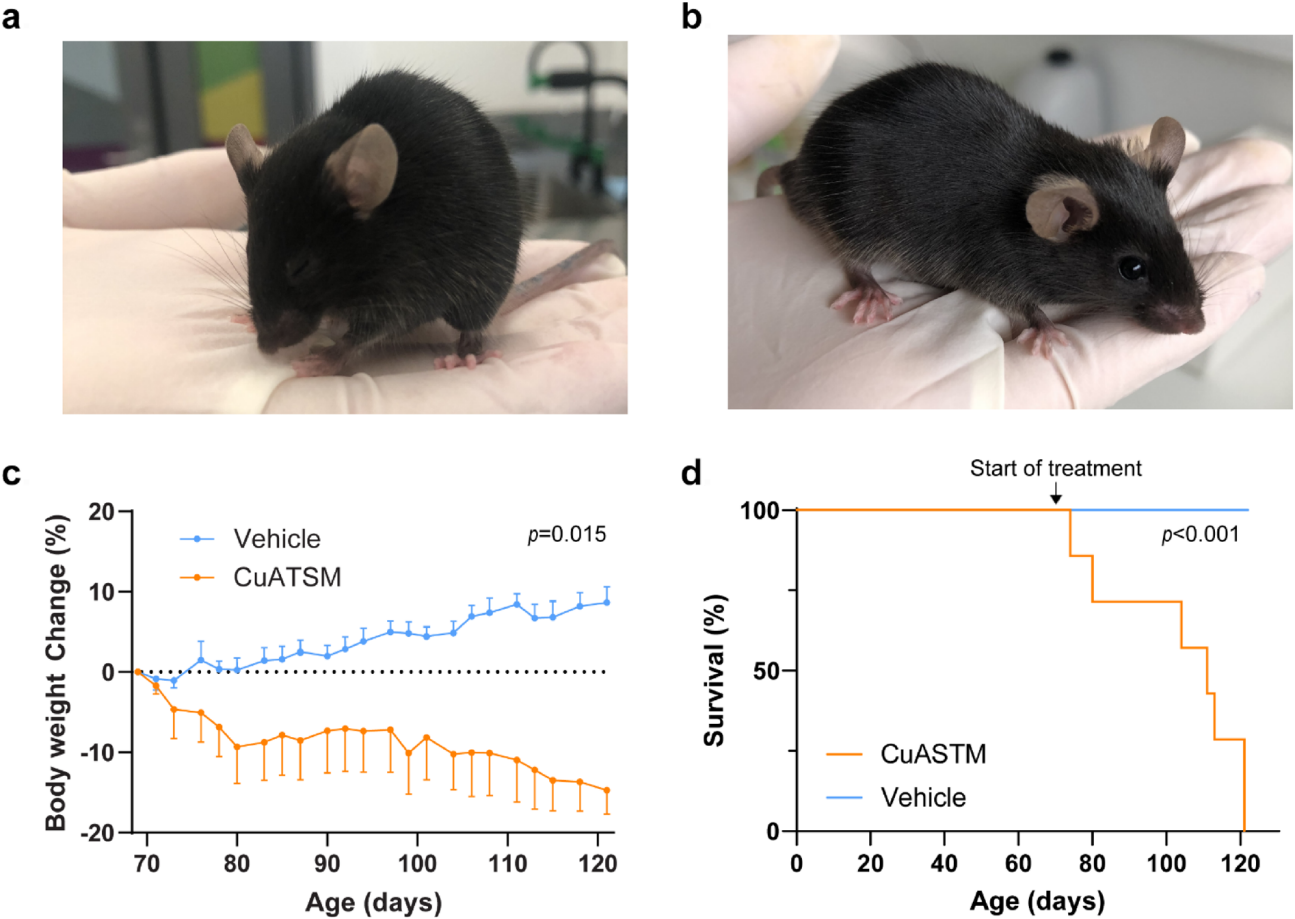
CuATSM (100 mg/kg/day) causes clinical signs of toxicity and weight loss in *SOD1*^*G93A*^ mice (maintained on a C57BL/6 background). *SOD1*^*G93A*^ mice were administered CuATSM (100 mg/kg/day) or vehicle via oral gavage. A subset of CuATSM-treated *SOD1*^*G93A*^ mice (7/20) developed clinical signs of toxicity including (**a**) hunched posture, orbital tightening, piloerection and low activity/respiration compared to vehicle-treated mice that exhibited, (**b**) typical posture and activity. (**c**) Percent body weight change of the subset of CuATSM-treated *SOD1*^*G93A*^ mice that developed clinical signs of toxicity compared to age- and sex-matched *SOD1*^*G93A*^ vehicle-treated mice and (**d**) survival of *SOD1*^*G93A*^ mice that exhibited toxicity following daily oral gavage administration of CuATSM (100 mg/kg/day) compared to age- and sex-matched *SOD1*^*G93A*^ vehicle-treated mice. Data represents mean $\pm \hspace{0.17em}$SEM (*n* = 7/treatment. Repeated measures ANOVA and Mantel–Cox tests were used to compare body weight change and survival, respectively, to compare relative differences between CuATSM- and matched vehicle-treated mice *SOD1*^*G93A*^ mice.

CuATSM (100 mg/kg/day) was seemingly tolerated for the majority of *SOD1*^*G93A*^ mice. However, seven CuATSM-treated mice (six male, one female) developed clinical signs of toxicity including; hunched posture, orbital tightening, piloerection, and low activity following CuATSM administration that were not observed in vehicle-treated mice (Fig. 1a,b; Table 2). The onset of observed clinical symptoms of toxicity was varied and ranged from 3–51 days following the commencement of CuATSM treatment. Furthermore, we observed CuATSM-treated mice produced dark brown/black faeces. *SOD1*^*G93A*^ mice typically exhibit weight gain until 110–130 days old^28,29^, when the onset of disease phenotype occurs, and gradual weight loss is observed thereafter. However, clinical symptoms observed in CuATSM-treated mice were accompanied by weight loss before 100 days old (Fig. 1c). A repeated measures one-way ANOVA of percentage body weight (compared to maximum weight prior to treatment) showed the subset of CuATSM-treated *SOD1*^*G93A*^ mice that exhibited signs of clinical toxicity progressively declined in percentage body weight from the commencement of treatment, while age-matched vehicle-treated *SOD1*^*G93A*^ mice increased over the same period (*p* = 0.015; Fig. 1c).

**Table 2:** Symptoms of CuATSM–associated toxicity, including weight loss, posture abnormalities and activity.

Symptom	Vehicle (n = 20)	CuATSM (n = 20)
Weight loss*	0	7
Hunched posture	0	6
Orbital tightening	0	5
Piloerection	0	5
Hypoactive	0	5

*Weight loss was defined as 10% body weight loss since beginning treatment.

To assess if the neurological score or motor coordination were negatively affected in the mice exhibiting clinical signs of toxicity, we analysed the neurological score and motor coordination in these mice compared to age-matched controls. The analysis showed no difference of neurological score, rotarod and pole test performance between CuATSM-treated mice exhibiting clinical symptoms and age-matched vehicle-treated mice (Supplementary Figure 1a–c, all *p* > 0.05), indicating CuATSM did not exacerbate ALS-associated motor symptoms in this subset of mice.

Once signs of either weight loss, hunched posture or orbital tightening were observed, mice were monitored closely. In all cases, the health of the mouse drastically declined within 96 h of observing any of the aforementioned symptoms and euthanaised by recommendation of the University of Wollongong’s animal welfare officer. A Mantel–Cox analysis of survival of CuATSM-treated mice at the dose of 100 mg/kg/day and subsequently euthanised due to observed clinical toxicity revealed a significantly reduced median survival compared to age-matched vehicle-treated *SOD1*^*G93A*^ mice (*p* < 0.001; Fig. 1d). The median survival for CuATSM-treated mice was 111 days (41 days following commencement of CuATSM (100 mg/kg/day) treatment), whilst all mice within the vehicle treatment group remained alive. Furthermore, to determine if CuATSM-induced toxicity was sex-dependent, a Fisher’s exact test was performed on male and female CuATSM-treated mice. Whilst there was a trend for male mice to be more susceptible to CuATSM-induced toxicity, this did not reach statistical significance (*p* = 0.057), likely due to the low sample size. Subsequently, CuATSM (100 mg/kg/day) treatment was ceased due to animal welfare concerns.

### Oral CuATSM administration (60 mg/kg/day) improved neurological function in *SOD1*^*G93A*^ mice

After observing clinical signs of toxicity and the subsequent euthanasia of a subset of CuATSM-treated mice, treatment was ceased in all mice. The remaining mice showed no signs clinical signs of toxicity following CuATSM 100 mg/kg/day treatment and were given a 1 week wash-out period where no treatment was administered in either group. Body weight, neurological score and motor coordination were continually assessed during this period. Following the 1 week wash-out period, mice recommenced CuATSM treatment at a new daily dose (60 mg/kg/day), and their body weight, ALS score and motor coordination were recorded. To avoid an age- or sex-bias, only vehicle-treated mice with age- and sex-matched CuATSM-treated counterparts were included in the remaining data used to determine the therapeutic efficacy of CuATSM treatment at 60 mg/kg/day. Due to the staggered age of the cohort, mice started to receive the lower 60 mg/kg/day CuATSM at various ages. The average age of mice upon recommencement of treatment was 112 days old for both the vehicle- and CuATSM-treatment groups (Supplementary Table 1). Furthermore, analysis of the remaining mice showed no significant difference in total body weight, percentage body weight change from pre-disease maximum and neurological score (p > 0.05; Supplementary Table 1) at the recommencement of CuATSM treatment (60 mg/kg/day).

**Figure 2:**
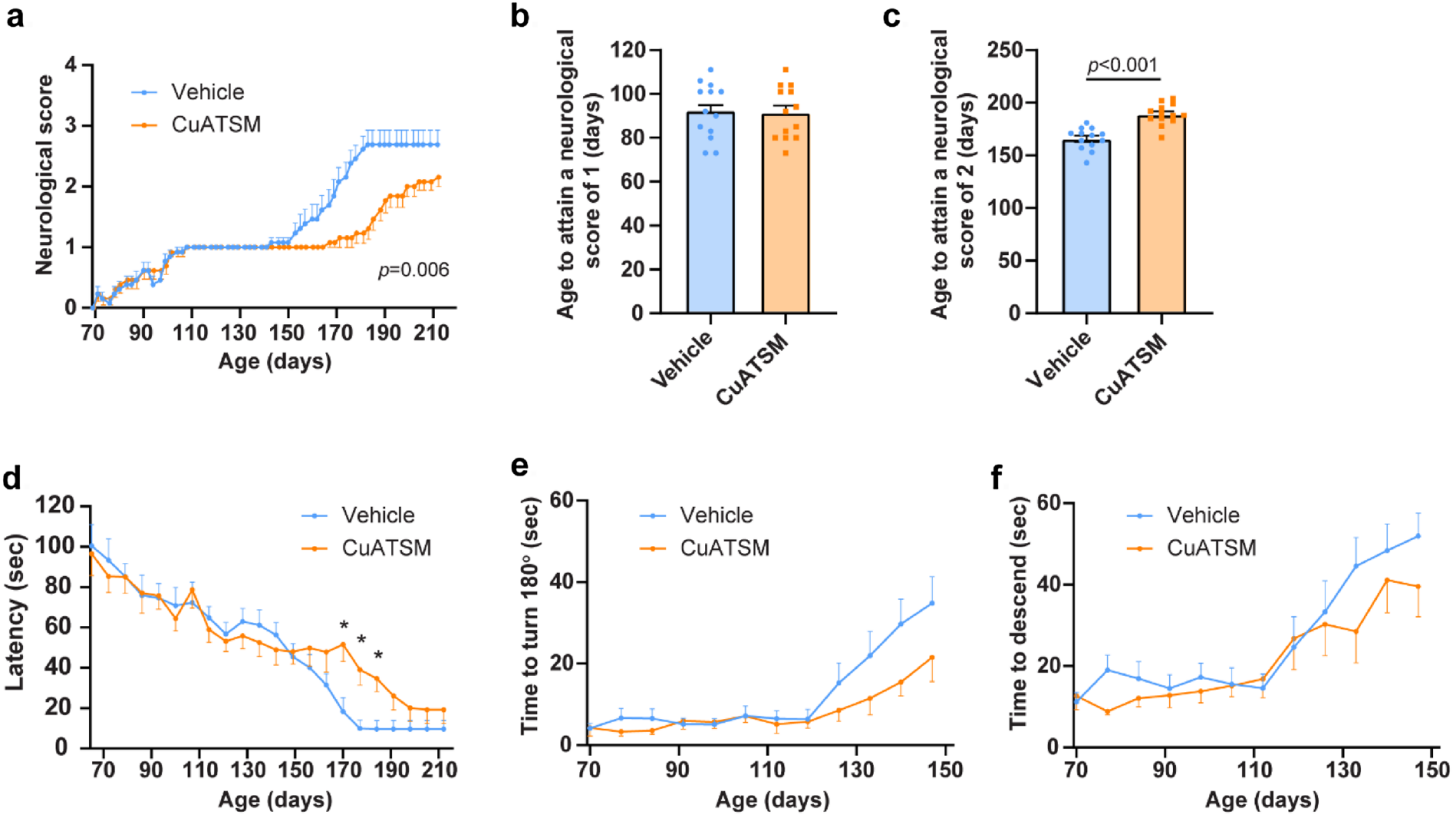
Oral CuATSM treatment slows disease progression and loss of motor coordination in *SOD1*^*G93A*^ mice. *SOD1*^*G93A*^ mice were administered CuATSM or vehicle via oral gavage. To assess neurological function, mice completed (**a**) Neurological scoring three times a week and the age to attain a neurological score of (**b**) 1 and (**c**) 2 was measured. In addition (**d**) rotarod and (**e**,**f**) pole test tasks were performed. Data are shown as mean ± SEM (*n* = 13/treatment). Repeated measures ANOVA were used to compare neurological score, latency, time to turn 180° and time to descend followed by post-hoc with Fisher’s least significance difference corrections. Independent *t* test were used to compare age to attain a neurological score of 1 and 2 between CuATSM- and vehicle-treated mice *SOD1*^*G93A*^ mice. *p < 0.05 compared to age- and sex-matched vehicle treated mice.

Assessment of neurological function was recorded three times a week by a blinded observer. Whilst onset of disease (neurological score = 1) was the same for both treatment groups (*p* > 0.05, Fig. 2b), repeated measures one-way ANOVA revealed CuATSM-treated mice exhibited a reduced rate of disease progression (*p* = 0.006, Fig. 2a), with CuATSM treatment delaying the mean onset of hind-leg paralysis by 23 ± 4 days (*p* < 0.0001, Fig. 2c). To further assess disease progression, mice performed weekly rotarod and pole test tasks. Mice from both treatment groups displayed a progressive decline in both rotarod and pole test performance (Fig. 2d–f). CuATSM treatment mitigated motor coordination decline on the rotarod, with CuATSM-treated mice having a significantly longer latency to fall from the rotarod when compared to vehicle-treated mice on days 170, 177 and 184 (all *p* < 0.05). Further assessment of motor coordination using the pole test task revealed whilst CuATSM-treated mice showed a general trend to turn 180° and descend the pole faster than vehicle-treated mice (Fig. 2e,f), no significant differences between treatment groups in the pole test were observed until 150 days old, when mice were unable to securely hold onto the pole and assessment was ceased.

### Oral CuATSM administration (60 mg/kg/day) reduced body weight loss and improved survival in *SOD1*^*G93A*^ mice

**Figure 3:**
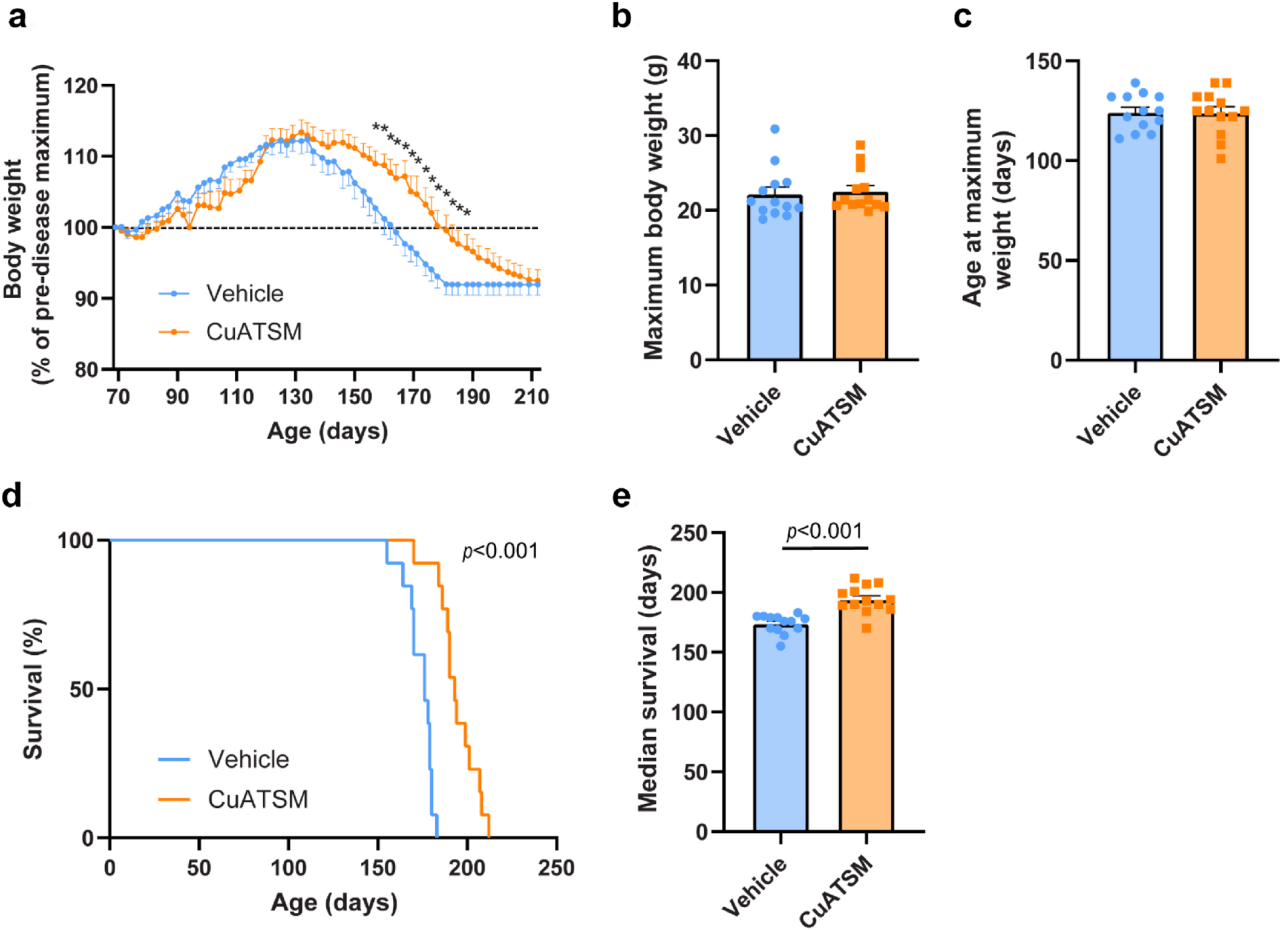
Oral CuATSM treatment delays weight loss and extends survival in *SOD1*^*G93A*^ mice (n = 13/treatment). *SOD1*^*G93A*^ mice were administered CuATSM or vehicle and (**a**) body weight recorded three times a week. (**b**) The mean age to reach peak body weight (+ SEM) and (**c**) maximum mean body weight (+ SEM) were measured. (**d**) A Kaplan–Meier curve of CuATSM- and vehicle-treated *SOD1*^*G93A*^ mice and (**e**) median survival (+ SEM) (*n* = 13/treatment). Repeated measures ANOVA were used to compare body weight change followed by post-hoc with Fisher’s least significance difference corrections. Independent *t* test were used to compare maximum body weight, age at maximum body weight and median survival between CuATSM- and vehicle-treated mice *SOD1*^*G93A*^ mice. *p < 0.05 compared to age- and sex-matched vehicle treated mice.

Body weight of CuATSM- and vehicle-treated *SOD1*^*G93A*^ mice was recorded three times a week throughout the duration of the study with both treatment groups reaching peak body weight at 124 days (Fig. 3a–c). Furthermore, there were no significant differences in peak body weight or the mean age at which maximum body weight was reached between treatment groups (*p* > 0.05, Fig. 3b,c). Both CuATSM- and vehicle-treated mice showed a gradual decline in body weight from 124 days onwards (Fig. 3a). Repeated measures one-way repeated ANOVA of body weight (percentage of pre-disease maximum) revealed a significant main effect of time (*p* < 0.001), but not significant treatment effect (*p* > 0.05). However, a significant interaction between factors was observed (*p* < 0.001), with post-hoc analysis showing CuATSM-treated mice slowed body weight loss and displayed a significantly higher percentage of maximum body weight between 155–188 days of age compared to vehicle-treated mice (all *p* < 0.05).

The humane-end stage was determined when mice displayed either a 20% weight loss compared to their maximum weight or an inability to right themselves within 30 s after being placed on their side. CuATSM treatment increased the survival of *SOD1*^*G93A*^ mice (*p* < 0.001), extending median end-stage by 9%, from 176 (vehicle-treated mice) to 193 days (*p* < 0.001, Fig. 3d,e).

### Oral CuATSM administration (60 mg/kg/day) increased SOD1 levels and activity

**Figure 4:**
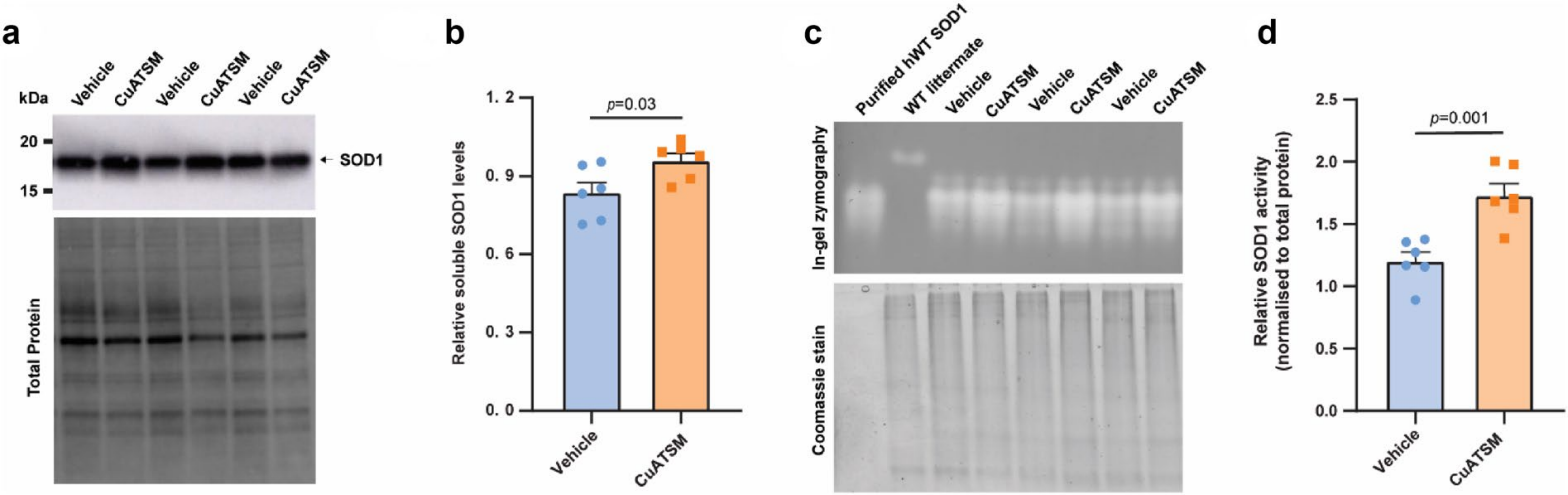
Oral CuATSM treatment increases soluble SOD1 levels and activity in the lumbar spinal cord of *SOD1*^*G93A*^ mice. (**a**) The relative levels of SOD1 protein were determined via western blot in (**b**). PBS-soluble fractions obtained from the lumbar spinal cord of vehicle- and CuATSM-treated mice. Quantification of relative SOD1 levels were normalised to total protein loading for each sample. (**c**) PBS-soluble homogenate from the lumbar spinal cord of CuATSM- and vehicle-treated mice were separated on a native 8% gel and SOD1 activity determined by in-gel zymography. Equal total protein amount across samples were determined by Coomassie signal. (**d**) Quantification of relative SOD1 normalised to total protein. Data shown are means ± SEM (n = 6/treatment). Data are from two independent experiments. Student’s *t* test was used to compare relative differences between CuATSM- and vehicle-treated mice *SOD1*^*G93A*^ mice. Full-length blots and gels are presented in Supplementary Figures 2 and 3, respectively.

CuATSM treatment has previously shown to increase soluble SOD1 levels and its enzymatic activity in the lumbar spinal cord of *SOD1* mice^17,18,20^. To investigate the effect of CuATSM on SOD1 levels, PBS-soluble and -insoluble fractions were prepared from the lumbar spinal cord of CuATSM- and matched-vehicle-treated mice. Analysis of SOD1 western blots revealed CuATSM-treated mice exhibited a 15% increase in PBS-soluble SOD1 in the spinal cord compared to vehicle-treated mice (*p* = 0.03, Fig. 4a,b).

To assess if SOD1 activity was increased in the lumbar spinal cord of CuATSM-treated mice, in-gel zymography was performed on the PBS-soluble fraction (Fig. 4c). Indeed, CuATSM-treated mice showed a 44% increase in SOD1 activity compared to vehicle-treated mice (*p* = 0.001; Fig. 4d).

## Discussion

Currently, CuATSM has emerged one of the most promising pharmacotherapeutic agents for the treatment of ALS. CuATSM has shown to delay the onset of motor impairment and prolongs survival in various ALS mouse models (Table 1), with no reported adverse events. Here, we sought to assess the therapeutic efficacy of a high dose of CuATSM (100 mg/kg/day) in high copy *SOD1*^*G93A*^ mice maintained on a congenic background (C57BL/6), a combination that had not been tested previously.

Results obtained from Phase I clinical trials observed that 8/14 patients receiving the highest dose of CuATSM (> 72 mg/day) exhibited reversible transaminitis^30^, consequently leading to the recommended Phase II dose set at 72 mg/day. In our *SOD1*^*G93A*^ model, administration of 100 mg/kg/day, across two doses a day by oral gavage led to morbidity and euthanasia in extremis for 7 of 20 treated mice after 3–51 days of treatment. It is unclear why only a subset of mice exhibited signs of toxicity, however we cannot eliminate the possibility that the remaining mice did not exhibit observable levels of pathological toxicity. To our knowledge, this is the first study to report adverse effects following CuATSM treatment. The signs of toxicity we observed, included weight loss, hunched posture and a hypoactive state, are consistent with symptoms reported previously in rodents given 100 or 200 mg/kg/day oral copper sulphate^31,32^ and intraperitoneal injections of copper lactate^33^. Histopathologic analyses of rodents given high doses of oral copper sulphate showed toxic effects on liver and kidney^31^. We were unable to confirm such effects on liver and/or kidney in CuATSM-treated mice that displayed signs of toxicity; histopathologic analyses on five of seven affected mice showed no significant lesions in liver or kidney (Supplementary Table 3). However, the albumin:globulin ratio and glomerular filtration rate, which are the most important markers for kidney disease, were not assessed. Moreover, of the seven CuATSM affected mice, we were able to obtain plasma samples for two, which subsequently showed elevated alanine aminotransferase (ALT) levels (Supplementary Table 4). Although, elevated ALT is excreted from both necrotic muscle and liver tissue. Differentiating between transaminases released from muscle and liver, requires testing for gamma glutamyl transferase (GGT), creatinine phosphokinase (CPK), and bilirubin, in addition to transaminases. Whilst, this information is important to provide a detailed understanding of the nature of the adverse events reported here, they remained beyond the initial scope of this work.

Although the clinical symptoms observed in a subset of mice treated with 100 mg/kg/day CuATSM displayed symptoms similar to previous reports of oral copper sulphate treatment in rodents, it is important to distinguish between mice treated with copper salts such as copper sulphate and CuATSM. The latter, is a charge neutral, stable, lipophilic compound with high membrane permeability and low redox potential (in the same range of redox potential as NADH), which is reduced by hypoxic but not normal mitochondria^34^. The complex remains stable when in copper(II) oxidation state but upon reduction to copper(I) the metal ion can be exchanged with high affinity copper(I) binding proteins. The reduction potential of CuATSM prevents reduction to copper(I) and subsequent release of copper in normal tissues^35^, however is selectively released in hypoxic cells or those with damaged mitochondrial electron transport chains^36^, as is the case in affected neuroglia cells in ALS. Under certain conditions, oxidation of CuATSM can lead to release of the metal ion^37,38^. Gastrointestinal microbiota secrete a diverse array of enzymes that metabolize orally administered drugs^39,40^, therefore oxidative metabolism in the gastrointestinal tract may lead to release of copper from CuATSM, but at this stage this is merely speculation and requires more detailed investigations.

There have been two previous studies which have utilised doses equal or higher than the 100 mg/kg/day dose initially used in the present study, without any reported adverse events^18,20^. However, it should be noted that the aforementioned studies utilised *SOD1*^*G93A*^ mice maintained on a B6/SJL background, unlike the present study where mice were maintained on a congenic C57BL/6 background, indicating toxicity may be strain-dependent. Strain-dependent differences in rodents have previously been reported in copper homeostasis and metabolism^41,42^. Furthermore, whilst there is a well-established strain difference regarding disease pathology of *SOD1*^*G93A*^ mice^43–46^, to our knowledge, there are no reports of pharmacotoxicology strain-dependent differences between *SOD1*^*G93A*^ mice maintained on different backgrounds. In addition, to potential strain-dependent tolerance differences, Williams et al.^18^ delivered CuATSM 200 mg/kg/day transdermally, in contrast to Hilton et al.^20^ and the present study, whereby CuATSM was administered orally. Bioavailability and pharmacokinetic comparisons between transdermal and oral gavage CuATSM delivery have not yet been performed and therefore it is not yet clear whether tolerability at 200 mg/kg/day via transdermal administration is the result of fundamental differences in pharmacological response or the fact that the 200 mg/kg/day study involved mice on the mixed B6/SJL background. It is also important to highlight that CuATSM is insoluble in aqueous media and the particle size of laboratory preparations may vary greatly from one study to the next due to variations from batch-to-batch within a laboratory or across laboratories. Thus, it is possible that different particle sizes may lead to differing dose-dependent absorption and saturation levels, and it is possible that high doses (100 mg/kg/day) delivered by oral gavage could lead to accumulation of the insoluble compound in the gastrointestinal tract. Although, the particle size of CuATSM used in this study was not assessed, histopathology assessment of affected mice showed no gastrointestinal abnormalities (Supplementary Table 3). Whilst we were unable to determine the underlying cause of toxicity, the present results from mice administered 100 mg/kg/day CuATSM provides important information for future preclinical work investigating CuATSM.

Despite, a subset of mice showing clinical signs of toxicity following 100 mg/kg/day CuATSM treatment, the remaining mice were subsequently given a 1 week wash-out period and administered 60 mg/kg/day CuATSM. These results support previous reports CuATSM has the capacity to delay disease progression and extend survival in *SOD1* mice (Table 1), particularly earlier intervention appears to lead to better therapeutic outcomes^18,23^, justifying our decision to begin treatment at a presymptomatic stage. We observed 60 mg/kg/day CuATSM treatment delayed weight loss, which has been attributed to muscle wastage^47^ and metabolic alterations^48^ in *SOD1*^*G93A*^ mice. A study performed by ALS Therapy Development Institute reported CuATSM slowed disease progression and provided a trend to extended lifespan in high copy number *SOD1*^*G93A*^ mice (maintained on a C57BL/6 background)^29^. The authors of the ALS Therapy Development Institute study acknowledged that their results reflected their use of a low daily dose (30 mg/kg/day) which had previously only produced statistically significant survival outcomes in low copy number *SOD1*^*G93A*^ mice or *SOD1*^*G37R*^ mice^17,23,24^. Dose–response studies have not been performed in high copy number *SOD1*^*G93A*^ mice. However, retrospective comparisons of CuATSM efficacy in high copy number *SOD1*^*G93A*^ mice suggests that increasing the dose improves outcomes. Williams et al.^18^ treated *SOD1*^*G93A*^ mice (maintained on a B6/SJL background) with 200 mg/kg/day from 50 days old, which resulted in increased mean survival of 19%, whilst Hilton et al.^20^ only reported a 9% increase when using 100 mg/kg/day. Notably, this apparent relationship between dose and survival in the high copy number *SOD1*^*G93A*^ mice came from two different studies which involved different methods for administering CuATSM (i.e., transdermal vs. oral). Collectively, multiple investigations utilising different mouse models of ALS and different methods of drug administration have shown that the therapeutic benefit derived from CuATSM relates to the dose of compound administered. Data presented herein for the dose of 60 mg/kg/day are supportive of this. However, our data presented for the higher dose of 100 mg/kg/day demonstrate that treatment beyond a maximum tolerated dose can be associated with serious adverse events. This highlights the importance of dose finding studies for tolerability, as per the successfully completed Phase 1 assessment of CuATSM in ALS patients are essential^30^.

Supportive of the growing body of preclinical and clinical evidence for the broad the therapeutic benefits of CuATSM, reports on the compound’s potential therapeutic mechanism of action appear diverse. There is substantial evidence that CuATSM increases copper bioavailability^36^, to improve physiological metalation and stability of SOD1, potentially benefiting patients with *SOD1* fALS^17,20,24^. Here, we were able to reproduce previous studies demonstrating CuATSM-treated mice display increased soluble SOD1 levels and activity^17,20,23^. Moreover, previous in vitro work performed by our group suggests CuATSM may provide greater protection against wild-type-like *SOD1* mutations and not metal-binding region (MBR) mutations^49^. These data raises questions about whether a copper bioavailability mechanism of action for CuATSM is suitable for patients carrying *SOD1* MBR mutations. The efficacy of CuATSM has yet to be tested in a mouse model carrying *SOD1* MBR mutations or a non-*SOD1* mouse model of fALS. However, there is ample evidence from numerous lines of investigation to illustrate that the therapeutic activity of CuATSM is not restricted to a mechanism of action that involves copper delivery to SOD1. For example, a recent pilot study reported CuATSM improves motor coordination and survival in a neurotoxin β-sitosterol β-d-glucoside model of sALS^50^. Whilst it is not known if SOD1 folding or copper homeostasis is altered in this model, evidence indicates that the therapeutic efficacy of CuATSM in this model may be through SOD1-independent mechanisms (reviewed by^51^). Alternative mechanisms may include scavenging peroxynitrite^23,25^ and anti-ferroptotic activity^52^, which are pathways implicated in both fALS and sALS.

Future work revealing pertinence of the mechanism(s) underlying the beneficial effects of CuATSM for both fALS and sALS will help further elucidate the disrupted molecular pathways in ALS. Furthermore, whilst the present study was not specifically designed to assess the toxicity of CuATSM, collectively, these initial results support the notion that high dose CuATSM may cause adverse effects, however further dose tolerance studies specifically addressing this issue are required.

## Materials and methods

### Animals

All research was approved by the Animal Ethics Committee (AE19/10) of the University of Wollongong (Wollongong, Australia) and complied with the National Health and Medical Research Institute, Australian Code of Practice for the Care and Use of Animals for Scientific Purposes. Mice hemizygous for the human *SOD1*^*G93A*^ transgene maintained on a C57BL/6 background (B6-Tg (*SOD1-G93A*)1Gur/j) were bred at the Australian Bioresources Animal Facility (Moss Vale, Australia) and housed in individually ventilated cages (IVC) (Type Mouse Version 1; Airlaw, Smithfield, Australia; air change: 90‐120 times per hour averaged; passive exhaust ventilation system). Mice were genotyped at the age of weaning (~ 3–4 weeks old) and *SOD1*^*G93A*^ transgenic mice (~ 4–5 weeks old) were transported and housed at the University of Wollongong in IVC cages (Greenline GM500, Techniplast, Australia) under a 12:12 h light–dark cycle (illuminated from 0700 to 1900 h). Mice were caged with littermates where attainable, with 2–4 females or 1–3 males per cage. IVC cages included a layering of Bed-O’Cobs™ corncob bedding (Techniplast, Australia), tissue, Bed r’Nest™ (Techniplast, Australia), a plastic house and a PVC tunnel. Food and water were available ad libitum. When mice reached 100 days old, water-soaked food pellets were placed on the cage floor and longer sippers placed on water bottles. Mice matched for date of birth and sex were equally divided into two treatment groups (*n* = 10/treatment/sex).

### CuATSM preparation and initial dosing regime

We chose to utilise CuATSM at 100 mg/kg/day in the present study based on a previous dose–response study which demonstrated a dose-proportional increase in life-span of *SOD1*^*G37R*^ mice up to 60 mg/kg/day^24^ and recent evidence demonstrating 100 mg/kg/day is therapeutically effective in *SOD1*^*G93A*^ mice (maintained on a B6/SJL background)^20^. CuATSM was synthesised as previously described^53^. CuATSM was suspended in a standard suspension vehicle (SSV; 0.9% w/v NaCl, 0.5% w/v Na-carboxymethylcellulose, 0.5% v/v benzyl alcohol, 0.4% v/v Tween-80) and sonicated via probe for five minutes immediately prior to treatment as previously described^29^. The CuATSM suspension was administered by oral gavage to *SOD1*^*G93A*^ mice twice daily (0830–0930 and 1730–1830) at a total dose of 100 mg/kg/day as previously described^20^. Equivalent volumes of SSV were administered to the vehicle-treated mice. Treatment commenced when mice reached 70 days of age.

### Adjusted CuATSM dosing regime

During the initial CuATSM treatment regime (100 mg/kg/day), clinical signs of toxicity were observed in a subset of CuATSM-treated mice following 3–51 days of treatment. Subsequently, treatment was ceased and remaining mice within the cohort (*n* = 13/treatment) were given a 1-week wash-out period and administered a lower dose of CuATSM (60 mg/kg/day) in a single daily treatment session (0830–0930) as previously described^24^. The revised dose of CuATSM (60 mg/kg/day) is the highest dose previously investigated and therapeutically effective in transgenic *SOD1* mice maintained on a C57BL/6 background. Previous work has also shown CuATSM at 60 mg/kg/day does not produce any toxic effects or weight alterations in wild-type C57BL/6 mice^24^. However, CuATSM at a 60 mg/kg/day dose has not been previously investigated in *SOD1*^*G93A*^ mice on a C57BL/6 background. Due to the staggered age of the cohort, mice started the adjusted treatment regimen (CuATSM; 60 mg/kg/day) at various ages (range = 80–125 days old), however the mean age, body weight and ALS score of mice within each treatment group were not significantly different once treatment recommenced (*p* < 0.05, Supplementary Table 1). The same CuATSM batch and drug preparation steps were not changed from the initial treatment regimen. Equivalent volumes of SSV were administered to the vehicle-treated mice.

### Weight and neurological score

Body weight was recorded three times a week, prior to the first daily treatment. Mice were also scored using the criteria outlined by the ALS Therapy Development Institute (TDI)^54^ (Supplementary Table 2) three times a week to assess neurological deficit. Scoring commenced at the beginning of treatment (70 days old) and was performed by observers blinded to treatment.

### Rotarod

The locomotor function of mice was assessed weekly, beginning at the first week of treatment (70 days old), using a five-lane accelerating rotarod (RotaRod Advanced, TSE Systems, Hesse, Germany). Mice were habituated to the rotarod assay 2 weeks prior to recording. Habituation sessions consisted of three acclimatisation sessions, with the first session run at a continuous 4 rotations per minute (rpm) for 180 s. The second and third habituation sessions were performed at an inclining speed of 4–20 rpm over a 180 s period. During the recording period (testing) the rotation speed of the rotarod was accelerated from 4 to 20 rpm over a 180 s period with the time taken to fail the task (latency to fall) recorded for each mouse. Mice were given three independent runs with a 30–60 s rest between runs. The maximum time each mouse was able to remain on the rod was recorded and included in the data analysis. To control for odour cues, the apparatus was cleaned with 70% ethanol after each trial.

### Pole test

Motor coordination, including grip strength, was also assessed through the use of the pole test with minor modifications as previously described^55^. Mice were placed facing upwards on a wooden vertical pole (diameter: 1 cm; length: 55 cm). Mice were given 60 s to descend the pole and complete the task. The time to turn 180° and subsequently reach the bottom of the pole was recorded. Mice who either did not turn around or slid down the pole with no active climbing were given a maximum score of 60 s. Each mouse was tested twice, with a 30–60 s rest between runs. The minimum time to descend the pole was recorded and included in the analysis. To control for odour cues, each apparatus was cleaned with 70% ethanol between each trial. All mice were assessed until 150 days old or until they were unable to be securely placed on the pole.

### Survival and end-stage

Mice treated with CuATSM (100 mg/kg/day) displaying signs of toxicity through weight loss, hunching, orbital tightening, and piloerection were indicators of a humane end-point and euthanasia determined by the University of Wollongong animal welfare officer. For mice treated with CuATSM (60 mg/kg/day) and matched vehicle-treated mice, disease end-stage was defined when the mice displayed either a 20% loss in maximum body weight or reached a clinical score of 4. Once end-stage was identified, mice were euthanised via asphyxiation using a slow-fill carbon dioxide technique, transcardially perfused with phosphate buffered saline solution (PBS) and lumbar spinal cords removed, snap frozen in liquid nitrogen and stored at − 80 °C.

### Tissue homogenisation and fractionation

Lumbar spinal cord (~ 30 mg) collected from PBS-perfused mice was homogenised in buffer containing PBS + 0.1% Triton-X100 supplemented with Halt™ protease and phosphatase inhibitors (ThermoFisher, Australia) with polypropylene pestles. Homogenates were centrifuged (20,000×*g* for 30 min at 4 °C) and the supernatant collected (PBS-soluble fraction). Protein concentration was determined via a DC assay as per the manufacturer’s instructions (Bio-Rad, Australia), and tissue homogenate stored at − 80 °C.

### Immunoblot

PBS-soluble fractions from the lumbar spinal cord (10 µg) were separated in stain-free TGX Any-kDa SDS-PAGE precast gels (Bio-Rad, Australia) under reducing conditions. Proteins were transferred onto 0.2 µm polyvinylidene difluoride membranes (GE Healthcare, Australia). Membranes were blocked in blocking solution containing 5% skim milk powder/Tris-buffered saline with 0.02% Tween 20 (w/v) for 1 h at room temperature (RT). Subsequently, membranes were incubated with anti-SOD1 antibody (Abcam, ab13498; 1:10,000) in blocking solution overnight at 4 °C. Following primary antibody incubation, membranes were incubated with horseradish peroxidase-conjugated secondary antibody for anti-rabbit IgG (Dako, P0448; 1:5000) in blocking solution for 1 h at RT. To visualise bands, membranes were incubated with ECL (GE Healthcare, Australia) and imaged with an Amersham 600RB Imager (GE Healthcare). SOD1 band quantification was achieved through the use of ImageJ software (Version 1.48, https://imagej.nih.gov/ij/)^56^ and intensity normalised to total protein and a pooled sample (containing equal amounts of each sample) to account for equal protein loading between samples and gels, respectively. Each sample was run in duplicate.

### In-gel zymography

SOD1 enzymatic activity was determined using in-gel zymography as previously described^57^. Briefly, lumbar spinal cord PBS-soluble fractions (2.5 μg) and 100 ng of purified recombinant human SOD1^WT^ protein was separated in 8% native PAGE gels. Subsequently, gels were incubated in 5 mM nitrotetrazolium blue chloride (Sigma-Aldrich) for 20 min with gentle agitation, before incubation in developer solution (10 mM tetramethylethylenediamine and 30 µM riboflavin) for 15 min. Gels were developed by exposure to fluorescent light until sufficient contrast between the achromatic zones and background was achieved and images captured using a calibrated densitometer (GS-900; Bio-Rad, Australia). SOD1 activity was quantified as absorbance of the clear bands using ImageJ software (version 1.48, https://imagej.nih.gov/ij/). Each sample was run in duplicate. To account for equal protein between samples, SOD1 activity was normalised to InstantBlue Coomassie stain (Sigma-Aldrich) from samples run on an adjacent gel.

### Data presentation and statistical analyses

For each data point, the mean ± standard error of the mean (SEM) is reported, unless otherwise indicated. Body weight, neurological score, rotarod- and pole test-activity were determined through repeated measures one-way ANOVA with Fisher's least significant difference test for comparisons between CuATSM- and vehicle-treated mice. Survival of CuATSM- and vehicle-treated mice was compared using a Mantel–Cox test. Student's *t* test was used to assess any other comparisons between CuATSM and vehicle treatment groups. To mitigate potential age- or sex-bias due to the attrition that resulted from treating with CuATSM at 100 mg/kg/day, only vehicle-treated mice with age- and sex-matched CuATSM-treated counterparts were included in the remaining data used to determine the therapeutic efficacy of CuATSM treatment at 60 mg/kg/day.

### Ethics declarations

All research was approved by the Animal Ethics Committee (AE19/10) of the University of Wollongong (Wollongong, Australia) and complied with the National Health and Medical Research Institute, Australian Code of Practice for the Care and Use of Animals for Scientific Purposes. The study was carried out in compliance with the ARRIVE guidelines.

## Supplementary Information

Supplementary Information.
