# Supplementary material for: CuATSM improves motor function and extends survival but is not tolerated at a high dose in SOD1G93A mice with a C57BL/6 background
Source: Sci Rep. 2021 Sep 29;11:19392. doi: 10.1038/s41598-021-98317-w (PMC8481268; doi:10.1038/s41598-021-98317-w)
Supplement: Supplementary file 1 — Supplementary Information. [file 41598_2021_98317_MOESM1_ESM.docx]

**CuATSM improves motor function and extends survival but is not tolerated at a high dose in *SOD1^G93A^* mice with a C57BL/6 background**

Jeremy S. Lum^1,2^, Mikayla L. Brown^1,2^, Natalie E. Farrawell^1,2^, Luke McAlary^1,2^, Diane Ly^1,2^, Christen G. Chisholm^1,2^, Josh Snow^2^, Kara L. Vine^1,2^, Tim Karl^3^, Fabian Kreilaus^3^, Lachlan E. McInnes^5^, Sara Nikseresht^4^, Paul S. Donnelly^5^, Peter J. Crouch^4^, Justin J. Yerbury^1,2^**^*^**

^1^Illawarra Health and Medical Research Institute, Wollongong, NSW, Australia

^2^Molecular Horizons and School of Chemistry and Molecular Bioscience, University of Wollongong, Wollongong, NSW, Australia

^3^School of Medicine, Western Sydney University, NSW 2560, Australia

^4^Department of Biochemistry and Pharmacology, University of Melbourne, Parkville, VIC 3010, Australia

^5^School of Chemistry and Bio21 Molecular Science and Biotechnology Institute, University of Melbourne, Victoria, Australia

***Corresponding author:**

Justin J. Yerbury, School of Chemistry and Molecular Bioscience, Faculty of Science, Medicine and Health, University of Wollongong, Wollongong, New South Wales 2522 Australia

Email: jyerbury@uow.edu.au

**Supplementary Materials**

| **Supplementary Table 1:** Characteristics of *SOD1^G93A^* mice at recommencement of treatment (CuATSM, 60 mg/kg/day) | | | |
| --- | --- | --- | --- |
|  | Vehicle | CuATSM | *p*-value |
| Age | 112 ± 4 | 112 ± 4 | >0.05 |
| Sex | 9F/4M | 9F/4M | - |
| Body weight (g) | 21.2 ± 0.7 | 21.7 ± 0.7 | >0.05 |
| Percentage body weight change from pre-disease maximum | 11.1 ± 1.6 | 11.1 ± 1.6 | >0.05 |
| ALS Score | 1 ± 0 | 1 ± 0 | >0.05 |
| Abbreviations: F, female; M, male; PN, postnatal day | | | |

| **Supplementary Table 2.** Amyotrophic lateral sclerosis neurological scoring system. Criteria outlined by the ALS Therapy Development Institute (Hatzipetros et al. 2015) | |
| --- | --- |
| Score | Criteria |
| 0 | Full extension of hind legs away from lateral midline when the mouse is suspended by its tail; mouse can hold this for 2 s, suspended 2-3 times |
| 1 | Collapse or partial collapse of leg extension towards the lateral midline (weakness) or trembling of hind legs during tail suspension |
| 2 | Toes curl under at least twice during walking of 12 inches, or any part of the foot is dragging along cage bottom/table |
| 3 | Rigid paralysis or minimal joint movement, foot not being used for generating forward motion |
| 4 | Mouse cannot right itself within 30 s after being placed on either side |

**
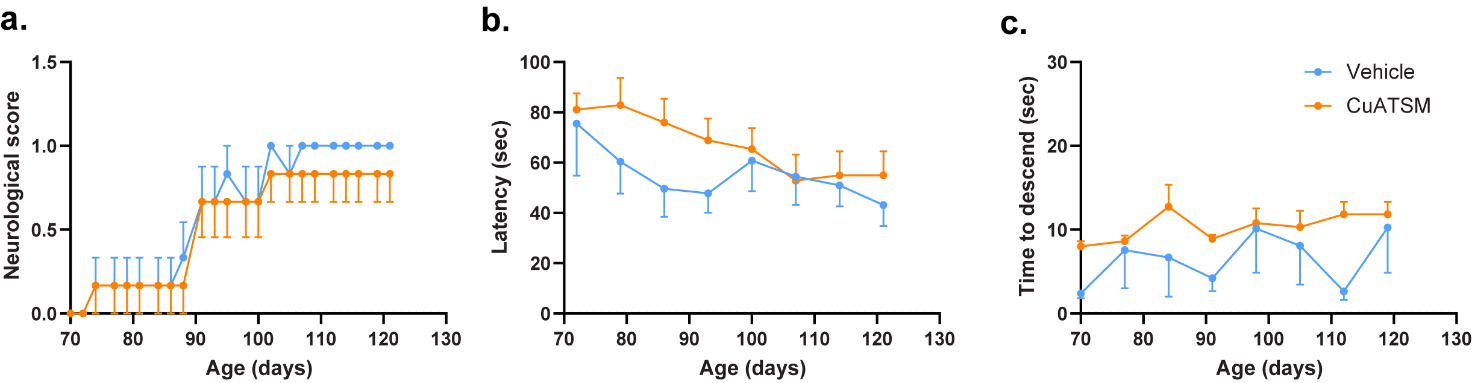
Supplementary Figure 1:** Oral CuATSM treatment (100 mg/kg/day) in SOD1^G93A^ mice exhibiting clinical signs of toxicity displayed no changes in neurological score or motor function in compared to age- and sex-matched vehicle-treated mice. To assess if CuATSM-treated mice exhibiting signs of clinical toxicity exhibited exacerbated or improved neurological function **a.** neurological scoring three times a week, in addition to **b.** rotarod and **c.** pole test task performance were analysed compared to age- and sex-matched vehicle-treated mice. Data are shown as mean ± SEM.


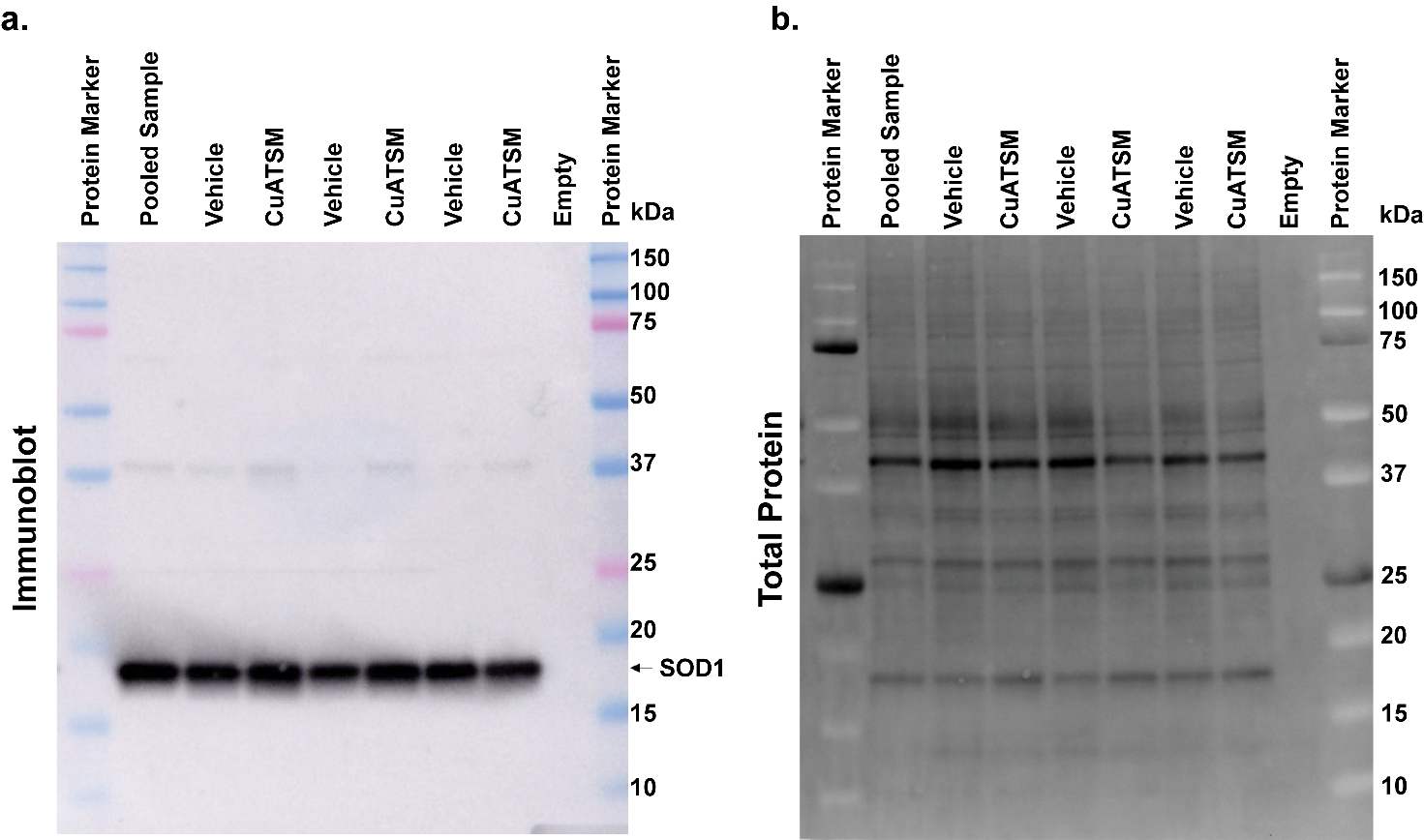


**Supplementary Figure 2.** Full length western blot images to accompany Figure 4a-b. To investigate the effects of oral CuATSM treatment on SOD1 levels in the lumbar spinal cord of *SOD1^G93A^* mice **a.** the relative levels of SOD1 protein were determined via western blot in PBS-soluble fractions obtained from the lumbar spinal cord of vehicle- and CuATSM-treated mice. Quantification of relative SOD1 levels were normalised to **b.** total protein loading for each sample and a pooled sample (containing equal amounts of each sample) to account for equal protein loading between samples and gels, respectively.


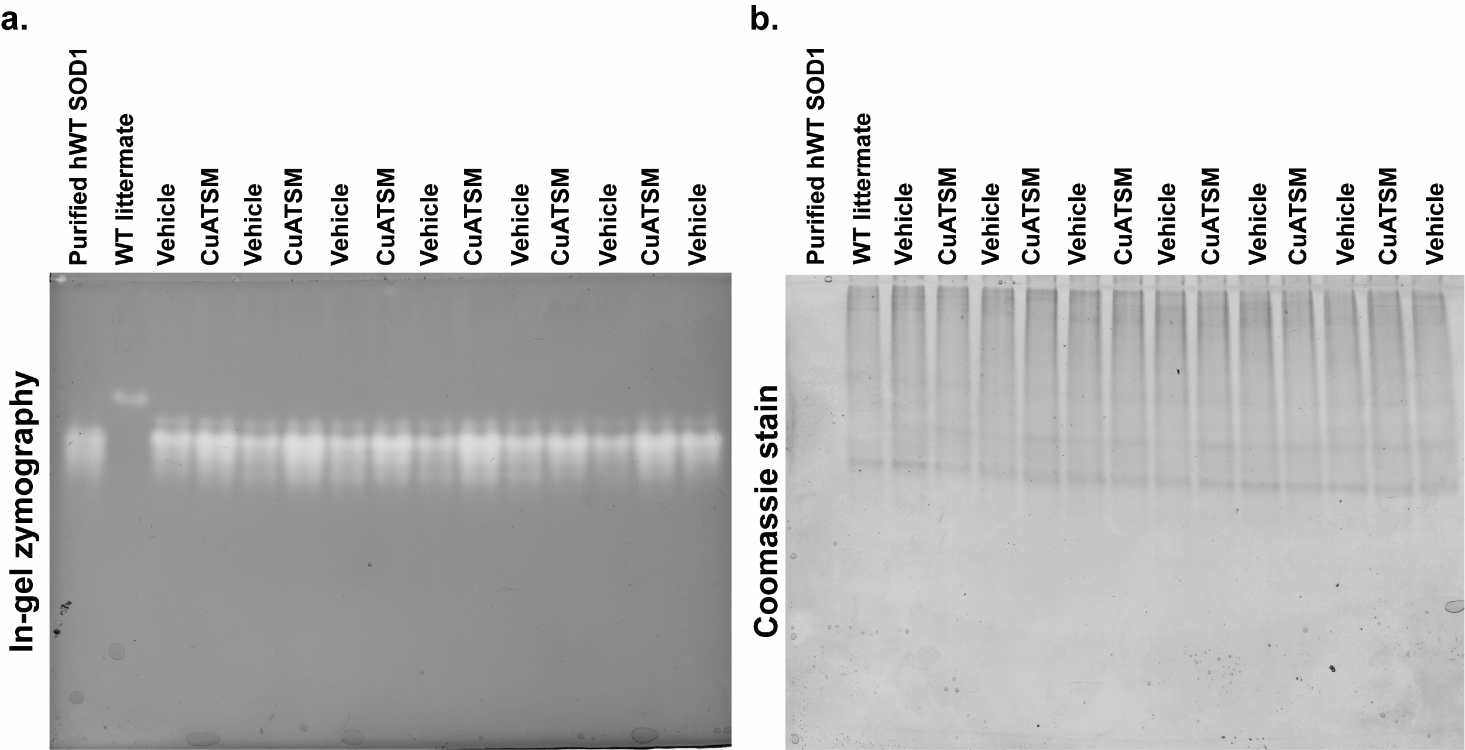


**Supplementary Figure 3.** Full length gel images to accompany Figure 4a-b. To investigate the effects of oral CuATSM treatment on SOD1 activity in the lumbar spinal cord of *SOD1^G93A^* mice **a** PBS-soluble homogenate from the lumbar spinal cord of CuATSM- and vehicle-treated mice were separated on a native 8% gel and SOD1 activity determined by in-gel zymography. Equal total protein amount across samples were determined by Coomassie signal. **b.** Quantification of relative SOD1 activity were normalised to total protein.

| **Supplementary Table 3:** Histopathology report obtained from CuATSM-treated mice exhibiting clinical signs of toxicity. Mice were euthanised at the recommendation of veterinary animal welfare officer via asphyxiation using a slow-fill carbon dioxide technique. Liver, kidney, spleen, pancreas and digestive tract were collected, stored in formalin, sectioned, hematoxylin and eosin stained and histopathology analysis performed by an external laboratory. | | | | |
| --- | --- | --- | --- | --- |
| **Mouse ID** | **Age** | **Gender** | **Tissues** | **Histopathology Report** |
| 4170 | 111 | M | Liver, pancreas, spleen, kidneys and heart | No significant lesions |
| 4160 | 113 | M | Liver, pancreas, spleen, kidneys and digestive tract | No significant lesions |
| 4108 | 121 | M | Liver, pancreas, spleen, kidneys and digestive tract | No significant lesions |
| 4197 | 80 | F | Liver | Moderate vacuolar hepatopathy |
|  |  |  |  | Minimal accumulations of lymphocytes in the portal tract |
|  |  |  |  | Patchy sinusoid and vascular congestion |
|  |  |  | Spleen | Multifocally, mononuclear phagocyte macrophages and periarteriolar lymphoid sheaths are mildly hyperplastic |
|  |  |  |  | Heterogenous populations of lymphocytes, plasma cells, macrophages, haemosiderophages and erythrocytes are interspersed |
|  |  |  |  | Mild accumulations of megakaryocytes, myeloid and erythroid precursors (extramedullary haematopoiesis). |
|  |  |  | Kidneys, pancreas, heart, lung and digestive tract | No significant lesions |

| **Supplementary Table 4:** Plasma biochemistry report obtained from CuATSM-treated mice exhibiting clinical signs of toxicity. Mice were euthanised at the recommendation of veterinary animal welfare officer via asphyxiation using a slow-fill carbon dioxide technique. Blood was obtained via cardiac puncture and plamsa sample biochemistry performed on a VetScan VS2 Chemistry Analyzer machine. | | | |
| --- | --- | --- | --- |
| **Mouse ID** | 4197 | 4157 | **Reference Range** |
| **Sex** | F | M |  |
| **Urea (mmol)** | 18.3 | >64.3 | 7.02-14.24 |
| **Creatinine (µmol/L)** | <18 | 180 | 16.94-20.21 |
| **Alanine aminotransferase (U/L)** | 659 | 278 | 18-82 |
| **Alkaline phosphatase (U/L)** | 67 | 124 | 66-176 |
| **Glucose (mmol)** | 2.1 | 1.5 | 8.88-11.82 |
| **Total protein (g/dL)** | 5.1 | 4.7 | 44.9-56.5 |
| Reference interval values are based on ^55,56^ | | | |
